# Literature Review of Occult Hepatitis B Infection and Hepatitis B Core Antibody-Positive Blood Donors

**DOI:** 10.7759/cureus.116625

**Published:** 2026-09-21

**Authors:** Aphisit Thongthaisin, Silvia Sauleda

**Affiliations:** 1 Academic and Continuing Education Department, National Blood Centre, Thai Red Cross Society, Bangkok, THA; 2 Laboratory for Transfusion Safety, Banc de Sang i Teixits, Barcelona, ESP

**Keywords:** anti-hbc positive donors, blood donation, blood donors, occult hepatitis b infection, transfusion transmitted infections

## Abstract

Hepatitis B virus (HBV) is one of transfusion-transmissible infection that is screened routinely in blood centers by serology and/or nucleic acid testing (NAT). Serology tests for HBV include antibody to hepatitis B core antigen (anti-HBc) and hepatitis B surface antigen (HBsAg). Blood centers around the world differ in their procedures for screening for HBV in blood donors. In high-income, HBV non-endemic countries, anti-HBc, HBsAg, and pooled NAT are usually used to screen blood donors, while in HBV endemic countries, anti-HBc is not usually used because it would lead to high discard rates. Donors who test positive only for anti-HBc have a history of HBV infection but have cleared the virus, resulting in negative results for both HBsAg and NAT. Deferral policy for anti-HBc-positive-only donors is restricted only to donors with high titers of anti-HBc and low titers of the protective antibody to hepatitis B surface antigen (anti-HBs) in some countries. In HBV endemic countries that screen for HBV by HBsAg and NAT, some blood donors test positive for HBsAg but negative for NAT (NAT yield donors). These test results can be the result of window period infection or occult hepatitis B infection (OBI), defined as the presence of replication-competent HBV DNA in the liver - in the presence or absence of HBV DNA in the blood - of individuals testing negative for HBsAg assessed by currently available assays. OBI donors can transmit HBV to recipients by blood transfusion. Therefore, in HBV endemic countries, individual donation NAT (ID-NAT) should be used to screen blood donors because it is more sensitive than pooled NAT and can detect both window period infection and OBI, while anti-HBc cannot detect window period infection.

## Introduction and background

Overview

Methods to Guarantee Blood Safety

Blood transfusion is a life-saving therapy, but it is not without risks. Transfusion-transmitted infection (TTI) is one of the well-known risks of blood transfusion therapy. The first case of viral TTI was reported in 1943. Laboratory screening for TTIs is done by serological methods since 1969, with hepatitis B surface antigen (HBsAg) being the first test that was implemented [1]. Transfusion-transmitted human immunodeficiency virus (TT-HIV) and hepatitis C virus (TT-HCV) crises in the 1980s and 1990s led to improved blood screening for TTIs based on precautionary measures [2]. There are three methods to guarantee blood safety, namely blood donor selection, laboratory testing, and pathogen reduction technology (PRT). For laboratory testing, the World Health Organization (WHO) recommended in 2009 that all donated blood must be screened at a minimum by serological methods: for HIV-1 and HIV-2 by either a combination of HIV antigen-antibodies or HIV antibodies, hepatitis B virus (HBV) by HBsAg, HCV by either a combination of HCV antigen-antibodies or HCV antibodies, and syphilis (*Treponema pallidum*) by specific treponemal antibodies [3]. However, serological methods have long window periods (time from infection to first reactive test): 19 days for HIV-1, 30 days for HBV, and 65 days for HCV [4]. For HIV, the aforementioned window period is of the fourth-generation HIV antigen/antibody test introduced in 1997, which is shorter than that of the previous generation antibody-alone immunoassays by four to five days due to the earlier detection of the p24 antigen [5]. TTIs can occur if the blood donor is in the window period and his or her blood is transfused to a patient. If using only serology, the risk of HBV transmission from transfusing blood from a donor in the window period is estimated to be 1 in 282,000 to 1 in 357,000 [1]. Therefore, nucleic acid testing (NAT) was later introduced in 1999 to complement serology, making the window period much shorter: 2.93 days for HIV-1, 10.34 days for HBV, and 1.34 days for HCV, if done by individual donor NAT (ID-NAT) [1,4]. Nowadays, the window-period residual risks of TT-HIV, HBV, and HCV in high-income countries and countries that utilize NAT are less than 1 in 1,000,000 units [2].

In addition to the problem of the window period, there are emerging and re-emerging infections, such as the SAR-CoV2 pandemic in 2020, for which screening tests may not be available, but may threaten blood safety. Therefore, the promotion of voluntary non-remunerated blood donors (VNRD) and blood donor selection are still essential. WHO recommends that “selecting VNRD from low-risk population for blood collection is the first step in reducing the risk of TTIs.” Blood donor selection is usually done by having potential donors respond to a validated questionnaire and confidential interview by trained personnel, which is dependent on the potential donors being honest about their health status and exposure to infections [6].

PRT offers additional safety measures to infectious units that elude blood donor selection and laboratory testing, particularly for new transmissible agents. An example of PRT in use in Thailand is psoralens/ultraviolet A (UVA) treatment for platelets and plasma. Psoralens intercalate between the bases of the nucleic acids of the pathogens, and upon illumination by UVA (320-400 nm), cross-links are formed, which prevent the replication and transcription of the DNA or RNA. However, PRT is expensive and therefore not implemented in every blood component in the developing world. Moreover, PRT methods for red blood cells (RBCs) and whole blood are still being developed, and there are concerns about neoantigen formation in the PRT-treated blood components and subsequent antibody production in the recipients [7].

Epidemiology of Transfusion-Transmissible Infections in Thailand

Thailand is an upper-middle-income country in Southeast Asia with a population of 66,052,615 as of 2023. The country is an endemic area for several of the aforementioned TTIs. It was estimated in 2016 that 759,000 individuals in Thailand are currently anti-HCV-positive, with 357,000 of them having viremic HCV infection [8]. It was estimated in 2019 that 470,000 individuals in Thailand were living with HIV, with more than half being men, and that there were 5,400 new adult HIV cases that year [9]. Syphilis is recognized as a major sexually transmitted infection in Thailand, as evidenced by its increased prevalence and co-infection with HIV [8]. Among blood donors, the prevalence of TTIs is lower: 0.068% for HIV 1 and 2, 0.210% for HCV, 0.447% for HBV, and 0.304% for syphilis in fiscal year 2022 [10].

HBV is a partially double-stranded DNA virus of the genus *Orthohepadnavirus*. It has 10 genotypes, namely A-J, with B and C being prevalent in Asia. It causes acute and chronic liver disease, cirrhosis, and hepatocellular carcinoma (HCC) if left untreated. Globally, there were approximately 245 million chronic HBV carriers in 2022 [11]. Thailand is an endemic area of HBV. In 2014, it was estimated that there were 2.2 million chronic HBV patients in Thailand [12]. Thailand started the HBV vaccination program for newborns in Chonburi and Chiang Mai provinces in 1988 before expanding to 12 provinces in 1990, and since 1992, a nationwide HBV vaccination program has been implemented as part of the Expanded Program on Immunization (EPI) until today. A study by Nilyanimit et al. in 2025 [11] estimated that 1.68% of the Thai population or 1.1 million people were chronic HBV carriers in 2024 with differences in the age groups: 0% in individuals aged less than 20, 0.3% in the age group of 21-30 years, 1.2% in the age group of 31-460 years, 2.1% in the age group of 41-50 years, 2.2% in the age group of 51-60 years, and 4.3% in those aged over 60, highlighting the program success after implementing for 32 years.

The Problems With Low Immunizers in HBV Infection

The problems with low immunizers in HBV infection gained attention in 1999 when a paper was published in the New England Journal of Medicine [13], showing the presence of HBV genomes in liver biopsy specimens from HBsAg-negative patients with chronic liver disease. This type of phenomenon is known as occult hepatitis B infection (OBI), which is defined as the presence of replication-competent HBV DNA in the liver - in the presence or absence of HBV DNA in the blood - of individuals testing negative for HBsAg assessed by currently available assays. It is classified into seropositive OBI, when antibody to hepatitis B core antigen (anti-HBc) and/or antibody to hepatitis B surface antigen (anti-HBs) is positive, and seronegative OBI, when both anti-HBc and anti-HBs are negative [14]. The prevalence of OBI in the general population is not well-defined, but the prevalence rate is higher in patients with chronic liver disease, including HBsAg-negative HCC (40-75%) and cryptogenic cirrhosis (32%). It is rarely found in blood donors, with a prevalence of 0-4.6% (median 1%) in HBsAg-negative-/anti-HBc positive-blood donors [14,15]. The clinical implications of OBI include transmissibility through blood transfusion and liver transplantation, HBV reactivation in conditions of strong immunosuppression, acceleration of the progression of chronic liver disease toward cirrhosis, and significant role in the development of HCC [15].

Regarding the eligibility of anti-HBc-positive OBI blood donors to donate blood, most countries do not allow them to donate blood due to the transmissibility of HBV to the recipients mentioned above, but some countries allow donors who have a high titer of the protective antibody anti-HBs (i.e., >200 IU/L in Japan, >100 IU/L in Germany and Austria) to be able to donate [16,17]. The Advisory Committee on the Safety of Blood, Tissues and Organs (SaBTO) of the UK included in their recommendations a discretion that anti-HBc-positive donors could be accepted if (1) they have adequate immunity to HBV, as demonstrated by an anti-HBs result of greater than 100 IU/L in a validated assay within 24 months of donation and (2) all of their donations will be tested by individual HBV DNA testing. However, this policy is not yet implemented in the UK [18].

Context

Methods to Guarantee Blood Safety From HBV in Thailand

Ensuring blood safety from HBV starts with blood donor selection. Deferral criteria related to HBV on the blood donor health questionnaire used in Thailand include the following: 1) had hepatitis after the age of 11 years (deferred permanently) 2) lived with a person who had hepatitis in the past 12 months, 3) ever been tested positive for hepatitis viruses (deferred permanently), 4) ever been imprisoned for more than 72 hours in the past 12 months, 5) received blood transfusion in the past 12 months, 6) been stuck by a bloody needle in the past 12 months, 7) had cosmetic procedures such as body/ear piercings, tattoo in the past four months, 8) ever been an intravenous drug user (deferred permanently), and 9) had high-risk sexual contact in the past four months [19].

In Thailand, HBV screening in blood donors is done by HBsAg and individual HBV DNA testing in every blood donation. In the UK, anti-HBc testing was added in 2022 for new donors only, while HBsAg and pooled HBV DNA testing are done in every blood donation. The rationale for adding anti-HBc to the blood donor screening program in the UK is to eliminate the risk of transfusion-transmitted HBV infection (TT-HBV) associated with OBI donors after seven cases of TT-HBV occurred in the UK during 2012-2022 from six OBI donors, some with severe clinical sequelae. The reason that the UK implemented anti-HBc instead of switching from pooled NAT to the more sensitive individual NAT is that it is found to be more economical. However, in high-endemic countries, including Thailand, it is not possible to implement anti-HBc screening due to the large number of the population that was exposed to the virus that would lead to a high deferral rate and low blood supply [20].

Clinical Implications of OBI Donors to Recipients

When screened with either of the two options mentioned above, OBI donors can be identified when the donors have HBsAg negative but HBV NAT positive and/or anti-HBc positive. Anti-HBc OB-positive donors in some countries can donate blood if the anti-HBs titer is high. The rationale for this policy is that it has long been recognized that anti-HBc-positive blood is not infectious when it contains more than 100 mIU/mL of anti-HBs. A prominent example is Japan, a country with an intermediate-high prevalence of HBV; the Japanese Red Cross (JRC) began nationwide screening for anti-HBc and anti-HBs in 1989 after encountering TT-HBV cases from HBsAg-negative/anti-HBc positive donors. To prevent serious blood shortages, anti-HBc-positive OBI donors with anti-HBs of 200 mIU/mL or more and/or low anti-HBc (titer < 1:32) are allowed to donate, excluding only donors with anti-HBs less than 200 mIU/mL and high anti-HBc (titer ≥ 1:32) [21,22].

However, in most countries, all blood donors who have been infected with HBV cannot donate blood, including anti-HBc-positive OBI donors. For example, in the USA, a non-endemic country, all HBV-infected donors, whether screened positive by serology or NAT, are indefinitely deferred. The rationale for this policy is that blood and blood products from OBI donors have been shown to transmit HBV, especially in the absence of neutralizing antibody. A study by the American Red Cross among 34.4 million donations during 2009-2015 found the rate of OBI to be 1.70 per 100,000 donations or 583 cases. Of these OBI cases, 92% (537/583) were identified by ID-NAT after the routinely used mini-pooled NAT (MP-NAT) of 6-16 samples failed to detect HBV, but anti-HBc was positive, leading to suspicion of OBI and subsequent ID-NAT testing. Of these 583 cases, plasma units could be retrieved in 80 cases, and 61% were found to have anti-HBs levels of less than 10 mIU/mL [23]. Therefore, it can be assumed that the majority of OBI donors are infectious to recipients. Another predictive factor of HBV transmission is the viral dose, with blood components that have more plasma volume being more likely to transmit HBV. A study by Allain et al. [24] found that the transmission rates by anti-HBs-negative OBI blood products were 85%, 51%, and 24% for recipients of fresh frozen plasma, platelet concentrates, and RBCs, respectively, with the 50% minimum infectious dose of OBI HBV DNA estimated to be at 1,049 (117-3,441) copies.

HBV Screening Algorithms of Blood Centers Around the World

An international survey on HBV screening algorithms was conducted by Fu et al. [25] among 30 blood establishments who were members of the International Society of Blood Transfusion (ISBT) working party on TTI diseases from 25 countries with data from 2022. Overall, 44% of the responding countries screen all donations by both anti-HBc and HBV NAT, including the USA, UK, Belgium, the Netherlands, Canada, Japan, France, and Germany. Moreover, 32% of the countries screen by HBV NAT, but not anti-HBc, including China, Russia, Australia, South Africa, Spain, Singapore, and Finland. Four countries (16%) do not screen by anti-HBc or HBV NAT, citing economic constraints or lack of resources, including Ghana, Iran, Nigeria, and Burkina Faso. Two countries (8%) screen by anti-HBc, but not HBV NAT, namely Turkey and Colombia.

According to the abovementioned survey, four respondents use anti-HBs titers to guide the deferral of OBI blood donors. Two respondents, namely Japan and the Netherlands, allowed DNA-negative donors with an anti-HBs titer of more than 200 IU/L to donate. Turkey allowed donation if anti-HBs titer was more than 100 IU/L. Lastly, Belgium temporarily deferred confirmed anti-HBc-positive donors with anti-HBs titer of 0 IU/L instead of permanent deferral if the titer was more than 0 IU/L, with an option for re-entry after the maximal period of six months if all HBV markers including anti-HBc are negative and anti-HBs titer remained at 0 IU/L [25]

Blood Donations in Thailand

The National Blood Centre (NBC) of the Thai Red Cross Society was established in 1969 to provide a safe and sufficient blood supply throughout the country. It consists of the NBC in Bangkok, 12 regional blood centers throughout the country, and one Plasma Fractionation Centre. Overall, 159 hospital blood banks, 8 in Bangkok and 151 in other provinces, are Blood Services Branches of NBC. In 2022, there was a total of 2,548,206 donations, accounting for 3.86% of the population, meeting the 1-3% target proportion of blood donors in a population to meet a country’s need from the WHO. Around 40% of blood donations in the country were done at NBC and its 12 regional blood centers throughout the country, while 60% were done at hospital blood banks. All blood donors in Thailand are unpaid blood donors who received no monetary compensation, dividing into 90-95% VNRDs and 5-10% family replacement donors, mainly in hospital blood banks in the provinces [10].

As mentioned above, NBC screens blood donations for HBV by HBsAg and ID-NAT. However, a substantial number of donations have discrepant results between the two methods, in other words, HBsAg negative but HBV NAT positive (NAT yield results). Specifically for HBV, there were 455, 449, 390, 292, and 266 NAT yield donations in the fiscal years 2018, 2019, 2020, 2021, and 2022, respectively. This can occur due to a window period infection, as HBsAg has a longer window period (30 days) compared to NAT (10.34 days), or it can be attributed to OBI donors.

Specific and Original Objectives

Because unnecessary deferrals lead to donor dissatisfaction and blood shortage, careful consideration of the scientific evidence regarding the rationale for donor deferral must be performed regularly. In an HBV endemic upper-middle income country, such as Thailand, adopting HBV screening algorithms and donor deferral criteria from non-endemic high-income countries that have high precautionary measures without modifications would lead to unacceptable blood shortages. Therefore, the general objective of the article is to provide a comprehensive literature review of the research on occult HBV and anti-HBc-positive blood donors in countries around the world. The specific objective of the first subsection is to provide a comprehensive literature review of the research on occult HBV and anti-HBc-positive blood donors in Thailand.

## Review

Material and methods

The searches for current peer-reviewed articles were conducted from March to April 2025 in the following databases: PubMed, Scopus, and Web of Science. Google Scholar was also utilized to locate open access articles. The search results were limited to articles that were written in English. No limit to publication date was applied. The main search concepts comprised terms related to “occult hepatitis B infection,” “blood donation,” “blood donors,” “transfusion-transmitted infection,” “nucleic acid testing,” “anti-HBs,” “anti-HBc positive donors,” and “HBsAg.” Variations of these terms were used to ensure exhaustive search results. The database searches were complemented with manual review of the reference lists of relevant articles, which resulted in a few additional articles being included in the review.

Results

Searching “Occult hepatitis B infection AND blood donation AND transfusion-transmitted infection AND anti-HBc positive donors AND anti-HBs AND nucleic acid testing AND HBsAg” on PubMed produced seven results (Table 1).

**Table 1:** Search results from PubMed anti-HBc, antibody to hepatitis B core antigen; DNA, deoxyribonucleic acid; HBsAg, hepatitis B surface antigen; HBV, hepatitis B virus

Title	First author	Journal	Year of publication	Bibliography
Prevalence of occult hepatitis B virus infection among the blood donors in Golestan province: cross-sectional study	Bahrami A	Iran J Microbiol	2022	[26]
Detection of occult hepatitis B and window period infection among blood donors by individual donation nucleic acid testing in a tertiary care center in South India	Keechilot CS	Pathog Glob Health	2016	[27]
Occult hepatitis B virus infection among Egyptian blood donors	Said ZN	World J Hepatol	2013	[28]
Evaluation of an individual-donation nucleic acid amplification testing algorithm for detecting hepatitis B virus infection in Chinese blood donors	Gou H	Transfusion	2015	[29]
Evaluating the cost-effectiveness of low-level HBV DNA screening in occult hepatitis B infection donors: A study from Shandong Blood Center, China	Chen J	Heliyon	2023	[16]
Serologic and molecular profile of anti-HBc-positive blood bank donors in an area of low endemicity for HBV	Kupski C	Dig Dis Sci	2008	[30]
Detection of an acute asymptomatic HBsAg-negative hepatitis B virus infection in a blood donor by HBV DNA testing	Weber B	J Clin Virol	2005	[31]

Searching “Occult hepatitis B infection AND blood donation AND transfusion-transmitted infection AND anti-HBc positive donors AND anti-HBs AND nucleic acid testing AND HBsAg” on Scopus produced four results. There was one duplicate, namely Chen et al. [16]. The search results excluding the duplicate are shown in Table 2.

**Table 2:** Search results from Scopus, excluding the duplicate study anti-HBc, antibody to hepatitis B core antigen; HBV, hepatitis B virus

Title	First author	Journal	Year of publication	Bibliography
Blood donor screening in the Netherlands: Universal anti-HBc screening in combination with HBV nucleic acid amplification testing may allow discontinuation of hepatitis B virus antigen testing	van de Laar TJ	Transfusion	2021	[32]
Occult hepatitis B virus infection and blood transfusion	Seo DH	World J Hepatol	2015	[21]
Hepatitis B virus infection among first-time blood donors in Italy: prevalence and correlates between serological patterns and occult infection	Romanò L	Blood Transf	2013	[33]

Searching “Occult hepatitis B infection AND blood donation AND transfusion-transmitted infection AND anti-HBc positive donors AND anti-HBs AND nucleic acid testing AND HBsAg” on Web of Science produced four results. All were duplicates, including Chen et al. [16], van de Laar et al. [32], Seo et al. [21], and Romanò et al. [33].

An additional open access article related particularly to Thailand [34] was found in Google Scholar by searching “Occult hepatitis B infection AND blood donation AND transfusion-transmitted infection AND anti-HBc positive donors AND anti-HBs AND nucleic acid testing AND HBsAg AND Thailand”. Another article related to OBI donors in Thailand [35] was located through the manual review of the reference lists of relevant articles and was included in the article.

Bahrami et al. [26] conducted a study including 4,313 HBsAg-negative blood donors selected randomly in Golestan province, which is the area with the highest prevalence of HBV in Iran (7.6-10.2%), in 2018. HBV markers, including anti-HBc (IgM and IgG), anti-HBs, and antibody to the hepatitis B e antigen (anti-HBe), were tested on these samples using the ELISA method. In addition, HBV DNA nested PCR, aspartate aminotransferase (AST), and alanine aminotransferase (ALT) were tested on these samples. They found that 384 (8.9%) of HBsAg-negative samples were anti-HBc-positive. Moreover, 302 (78.65%) of these samples were anti-HBs-positive, with 118 (30.75%) and 184 (47.9%) of them having antibody titers between 10 and 100 IU/mL and between 100 and 600 IU/mL, respectively, and 152 (39.6%) of anti-HBc-positive donors were anti-HBe-positive. HBV DNA was not detected in any samples. Thirty-nine (0.90%) samples were anti-HBc positive but negative for both anti-HBs and anti-HBe. These isolated anti-HBc donors were re-tested after one year and showed the same results of anti-HBc positive alone. Their AST and ALT levels (19.59 ± 6.61 U/L and 22.64 ± 7.8 U/L, respectively) were not statistically different from those of the anti-HBc-positive donors (21.43 ± 6.50 U/L and 22.09 ± 9.27 U/L, respectively). Therefore, the authors concluded that confirmatory testing for anti-HBc should be done in these isolated anti-HBc samples to avoid deferring blood donors based on false-positive anti-HBc results and that anti-HBc testing with ID-NAT should be performed in areas with moderate prevalence of HBV, such as Iran, to increase blood safety.

Keechilot et al. [27] screened 24,338 blood donors in a tertiary care center in South India from 2014 to 2015 for HBsAg, anti-HIV, and anti-HCV, with all serologically negative donors being later screened by ID-NAT for viral DNA and RNA. NAT-positive samples were tested with discriminatory NAT (dNAT), and all samples positive for HBV NAT were tested for anti-HBc, anti-HBs, and HBeAg. Forty-six (0.19%) donors from 24,338 donors screened were HBsAg-positive. ID-NAT and dNAT testing of HBsAg-negative donors identified five (0.0205%) donors who were positive for HBV DNA. Four of the samples were positive for anti-HBc and thus classified as true OBI donors. One sample was negative for both anti-HBc and anti-HBs and was classified as a window period donor. Among the four OBI donors, three (75%) were anti-HBs-positive (antibody titer ≥10 IU/L). All four OBI donors were HBeAg-negative. The authors concluded that anti-HBc was not suitable for India because it is only appropriate in low endemicity settings (<2% anti-HBc positivity) due to the discard rates, it cannot detect window period infection, and NAT-based screening for all HBsAg-negative donations should be implemented instead.

Said et al. [28] published a study on OBI blood donors in Egypt in 2013 by screening 3,167 serologically (HBsAg, anti-HIV, and anti-HCV) negative blood donors for anti-HBc, and additionally testing their AST and ALT levels. Positive anti-HBc samples were tested for anti-HBs titers. HBV DNA was tested by real-time PCR in some anti-HBc-positive samples. Recipients of these blood components were followed up at three to six months for AST, ALT, HBsAg, anti-HBc, anti-HBs, and HBV DNA. They found that 525 (16.6%) of 3,167 blood donors were positive for anti-HBc and that 64% of them were anti-HBs-positive, including 92 (27.8%) with a titer of 10-99 IU/L, 127 (38.4%) with a titer of 100-999.9, and 112 (33.8%) with titer ≥ 1,000 IU/L. However, confirmatory tests for anti-HBc with a different technique were performed in 498 samples and confirmed 451 (90.6%) of them, giving a prevalence of 14.2% for anti-HBc positivity among blood donors. HBV DNA was quantified in 303 anti-HBc-positive samples, and 52 (17.2%) of them were detected. Forty-nine percent had a viral load of <200 IU/mL, signifying low level of viremia that correlates with chronic resolving infections. Age above 30 and marriage were found to be associated with positive anti-HBc, with adjusted odds ratios of 1.8 (1.4-2.4) and 1.4 (1.0-1.9%), respectively. Interestingly, 34 out of 216 recipients came to follow-up, and their follow-up samples were negative for all HBV markers and HBV DNA. No one developed post-transfusion hepatitis. Eleven of the 34 recipients received blood from anti-HBc-confirmed positive donors: nine (81.8%) were HBV DNA-negative and two (18.2%) were HBV DNA-positive, with HBV DNA concentration of 8 IU/mL in one donor and 3.3 × 10^4^ IU/mL in the other donor.

Gou et al. [29] conducted a multicenter study in 10 blood centers in China during 2010-2012 to evaluate the efficiency of a multiplex ID-NAT (Ultrio assay) and discriminatory testing algorithms for detecting HBV in Chinese blood donors. In total, 1,205,796 HBsAg-negative donors were tested by ID-NAT, and positive samples were subsequently tested with quantitative PCR (qPCR), HBsAg, anti-HBc, HBeAg, anti-HBe, and anti-HBs. In addition, a control group of 4,317 ID-NAT-negative samples was tested for anti-HBs and anti-HBc. The results showed that 2,033 (0.17%) of all donations were multiplex ID-NAT reactive, and among further testing of 1,776 samples, 548 (30.9%) were HBV discriminatory assay (dHBV)-reactive. About a quarter (31.1%) of HBsAg-negative, multiplex ID-NAT-positive, and dHBV-negative samples were anti-HBc-positive and anti-HBs-negative, signifying potentially infectious low-viral-load OBI donors. The authors discussed that anti-HBc screening is not possible in China because it would lead to 36% discard rates. They found that 81.8% of multiplex ID-NAT-positive and dHBV-negative donations of anti-HBc-positive donors had negative anti-HBs or anti-HBs titer of less than 100 IU/L. Therefore, the authors concluded that ID-NAT repeat testing to increase the probability of identifying low-viral-load window period and OBI donations is more practical than including anti-HBc in the screening algorithms to enable release of NAT non-repeat-reactive donations because it would not identify low-viral-load window period and complicate procedures.

Chen et al. [16] conducted a study in one blood center in China among 220,445 HBsAg-negative blood donors during 2018-2022. The samples were tested by the 6-sample minipool NAT (MP-NAT) method, and NAT-positive samples were subsequently tested for anti-HBc and anti-HBs. Anti-HBc-positive samples were later tested by ID-NAT, and any ID-NAT-positive samples were confirmed by an alternate NAT method. They found that the positivity rate of HBV DNA detection was 0.031% (69/220,445) by MP-NAT. Among 67 MP-NAT-positive samples, 55 (82.09%) and 25 (37.31%) samples were positive for anti-HBc and anti-HBs, respectively. The anti-HBs titers were 10-20 IU/L, 20-100 IU/L, 100-200 IU/L, and 200-500 IU/L in 6 (8.96%), 12 (17.91%), 4 (5.97%), and 3 (4.48%) donors, respectively. Moreover, 18.95% (909/4,797) were positive for anti-HBc among randomly selected HBsAg-negative and MP-NAT-negative donors. Further ID-NAT testing was done in randomly selected 164 of these donors and found to be positive in two (1.22%) donors, resulting in a detection rate of OBI of 0.22% (1.22 × 0.1895) among HBsAg-negative and MP-NAT-negative donors. The authors discussed that due to financial constraints, their blood center could not implement ID-NAT in all donations. Therefore, they recommended that anti-HBc be performed instead and that ID-NAT be performed only on anti-HBc-positive donors. Plasma-containing products from anti-HBc-positive donors with low or negative anti-HBs should be discarded. Moreover, they recommended that anti-HBs and anti-HBc should be tested in recipients, and recipients with low or negative anti-HBs should not be transfused with plasma-containing products from anti-HBc-positive donors.

Kupski et al. [30] conducted a study in an HBV low endemic region of the southern region of Brazil, which had a seroprevalence of 0.5-1% between 2003 and 2005. Overall, 244 blood donors who were screened positive for total anti-HBc but tested negative for HBsAg on routine testing were further tested for anti-HBs, HBeAg, anti-HBe, and HBV DNA. They found that 209 (85.7%) donors were immunized for anti-HBs, including 36 (14.8%) donors with anti-HBs titer of ≥10 to <100 IU/L and 173 (70.9%) donors with anti-HBs titer of ≥100 IU/L. They tested 164 samples for HBeAg and anti-HBe. All of them were negative for HBeAg, while 109 (66.5%) samples were anti-HBe-positive. The authors found a statistically significant association between anti-HBe-positive individuals and strongly positive anti-HBs titer of ≥100 IU/L (p = 0.026). All 241 (100%) blood samples that were tested for HBV DNA were negative. The authors discussed that the majority of exclusively total anti-HBc-positive donors included in this study were immunized as a result of previous exposure to HBV (anti-HBs-positive), had no detectable viral replication (HBeAg-negative), and had eliminated the virus spontaneously (HBV DNA-negative). Therefore, to prevent the high discard rate in times of blood shortages, the authors suggested that measures should be taken to facilitate the use of these blood units in HBV low endemic areas.

Weber et al. [31] published a case report on a blood donor whose donation tested positive for HBV in a pool of 96 donations in 2001 in Austria. The donor was a repeat asymptomatic female donor who tested negative for serological markers of HBV (HBsAg, anti-HBc), HIV, and HCV and had liver enzyme values in the normal range. One week later, total anti-HBc and anti-HBc IgM were positive, and thus acute HBV infection was suspected. At the third follow-up 45 days later, total anti-HBc was the only positive marker. Anti-HBs became positive with a titer of 12 IU/L and 10 IU/L on the fourth and final follow-up at three months and two years later, respectively. No HBsAg and HBV DNA were detected in all the four follow-up samples. HBeAg and anti-HBe were negative in all the five times of testing, including the day of donation. This case demonstrated asymptomatic self-limiting acute HBV infection with consecutive immunity in an immunocompetent adult. HBsAg was present at low concentrations and during a very short time, and thus it was not detected by screening in this blood donor. The authors suggested HBV DNA screening with a sensitive assay based on the findings in this case so that not only acute HBV infection similar to this case would be detected but also OBI in chronic carriers would be detected. Moreover, anti-HBc screening would also reduce the residual risks in chronic isolated anti-HBc-positive donors who usually have a low viral load and therefore failed to detect HBV DNA.

van de Laar et al. [32] provided a flow chart of anti-HBc-based deferral in blood donors at Sanquin in the Netherlands. Each donation in the Netherlands is screened for HBsAg, anti-HBc, and HBV DNA in MP-NAT of six donations. In 2011, universal HBV vaccination of newborns began, and anti-HBs-dependent anti-HBc screening algorithm in blood donors was implemented. This algorithm retained the eligibility of new donors or repeat donors who tested for anti-HBc for the first time, and the result was repeat reactive if their anti-HBs was ≥200 IU/L because anti-HBs titer below 200 IU/L is considered at risk for chronic OBI. However, repeat donors who used to test negative for anti-HBc and seroconverted (suggesting recent HBV exposure) are permanently deferred if anti-HBs is ≥10 IU/L but are allowed to return for testing at six months (temporarily deferred) if the anti-HBs is undetectable. Upon return, if anti-HBc is positive, the donor is permanently deferred irrespective of anti-HBs titer. This so-called two-strike policy was implemented to circumvent permanent donor loss due to one-time false-positive anti-HBc reactivity, which is associated with the absence of anti-HBs.

Moreover, van de Laar et al. [32] conducted a study since the implementation of anti-HBc screening in 2011, as described above, until its replacement by an alternative anti-HBc screening test in 2018 in all visits in the Netherlands (n = 5,603,407) to evaluate each of the three screening tests (HBsAg, MP-NAT, and anti-HBc) for its added value for intercepting potentially infectious and/or recent HBV infections. They found that over the study period, 89 donors were identified as HBV-infected by at least two of the three screening tests. Single marker yield was as follows: 5 MP-NAT-only, 0 HBsAg-only, and 20 anti-HBc-only donors with confirmed or probable recent HBV infection based on determination of an imputability score (≥3), i.e., ID-NAT-positive (5 points), detectable core IgM (1 point), anti-HBc seroconversion (1 point), high anti-HBc reactivity (1 point), anti-HBs seroconversion, or a rise in anti-HBs titer ≥200 IU/L (1 point). Moreover, 1,067 potentially infectious donors who were at increased risk for OBI were identified by high or “true” anti-HBc reactivity (S/CO ratio < 0.50). Based on the two-strike policy of anti-HBc reactive donor deferral aforementioned, the permanent deferral rate was 0.45% (2,627 donors) from the initial deferral rate of 0.51% (2,977 donors), saving 350 anti-HBs-negative donors with one-time anti-HBc false reactivity. The authors concluded that HBsAg is redundant in the presence of universal anti-HBc screening because it did not intercept infectious donations from donors with recent infection or OBI. Moreover, the authors discussed that due to the low infectious dose of HBV, switching from MP-NAT plus anti-HBc screening to ID-NAT will reduce TT-HBV.

Seo et al. [21] published a review article on OBI and blood transfusion in 2015 in which they summarized the role of blood screening for HBV and updated the known risks of OBI transfusion-transmission. The anti-HBc test was first introduced for blood screening in the mid-1980s in non-endemic countries. The prevalence rates of anti-HBc-positive blood donors vary among countries depending on the prevalence in the population of that country, including 0.23%, 0.56%, 0.70%, 1.1%, 1.88%, 4.85%, 10.82%, 13.5%, 14.2%, 14.9%, and 17.28% among blood donors in United States, the United Kingdom, Denmark, Japan, Germany, Italy, India, South Korea, Egypt, Greece, and Pakistan, respectively. Studies have shown an HBV DNA positivity rate of 0-15% among these anti-HBc-positive-only donors. HBV NAT testing was first introduced in Germany in 1997. The prevalence of OBI among blood donors was estimated to be 8.55 per 1 million donations according to an international survey on NAT testing. OBI is transfusion-transmissible, although the risk is low. OBI test results (HBsAg negative, HBV NAT positive) can be caused by (1) the window period, (2) the tail-end stage of chronic hepatitis B, (3) low-level viral replication after recovery from hepatitis, and (4) escape mutants not detected by current HBsAg tests. Studies have shown that the transmission rate during the window period was 10 times higher than the transmission rates from OBI donors with low-titer anti-HBc. A study in Taiwan identified 12 (0.11%) OBI donors in 10,824 repository samples, but no TT-HBV occurred among the recipients, suggesting that the risk of TT-HBV is lower in endemic countries, as most recipients have already encountered the virus. Finally, the authors discussed that despite the higher costs, HBV NAT is more suitable than anti-HBc for screening in endemic areas of HBV because it can detect window period infections.

Romanò et al. [33] conducted a prospective study in 21 blood centers in Italy over a period of one year, April 2004 to March 2005, in 31,190 first-time blood donors who were not in the age groups that received universal HBV vaccinations, accounting for 12% of all yearly donations in the country. The donors were tested for HBsAg and anti-HBc. Among donors tested positive in either of these tests, HBV DNA, HBeAg, anti-HBe, anti-HBs, and anti-HBc IgM were also tested. They found that 100 (0.32%) donors were positive for both HBsAg and anti-HBc, 2 (0.01%) donors for HBsAg alone, and 2,593 (8.3%) donors for anti-HBc alone. Among the last group of anti-HBc-positive-alone donors, 2,436 (93.9%) samples were tested for other HBV markers and found that 1,152 (47.3%) samples were anti-HBs-positive, 960 (39.4%) samples were anti-HBs- and anti-HBe-positive, 253 (10.4%) samples were anti-HBc-positive only, and 71 (2.9%) samples were anti-HBe-positive. Among the anti-HBs-positive (titer >3 IU/L) donors, the titers were below 10 IU/L, 10-100 IU/L, and more than 100 IU/L in 5.4%, 30.7%, and 63.9% of the donors, respectively. HBV DNA testing was done in 89.7% (2,186 samples) of this anti-HBc-positive-alone group and found 12 (0.55%) HBV DNA-positive samples consistent with a diagnosis of OBI. Moreover, a statistically significant difference in HBV DNA positivity rate between the anti-HBs-negative (1.68%) and anti-HBs-positive (0.37%) groups was observed. The HBV DNA positivity rate among the anti-HBs-negative and anti-HBe-positive subgroup was highest at 7.02%. Therefore, the authors concluded that the frequency of OBI among Italian first-time blood donors is 1 in 2,599 donors (12 in 31,190 donors) and that HBsAg, HBV DNA screening, and HBV vaccinations are essential to reduce the risk of TT-HBV.

Chimparlee et al. [34] published a review article on HBV and HCV in Thai blood donors in 2011. They provided the timeline of the improvement of measures to decrease TT-HBV and TT-HCV. Firstly, regarding donor self-selection, all blood donations at NBC have been done by VNRDs since 1985. Prisoners were excluded in 1986. Secondly, regarding screening tests, only donations from new blood donors were screened until 1986, when all donations were screened, which was done solely by serological tests until the addition of NAT in 2006.

Moreover, as mentioned earlier, nationwide HBV vaccination program was implemented in 1992 for all newborns at zero, one to two, and six months. Hepatitis B immunoglobulin (HBIG) was also given to newborns of HBV carrier mothers. The national coverage of HBV vaccine was 77.4%, 88%, 93.4%, 95.4%, 96%, and 98.3% in 1994, 1995, 1996, 1999, 2003, and 2008, respectively. Therefore, the authors concluded that the drastic change of HBV prevalence in Thai first-time blood donors from 7.14% in 1988 to 2.63% in 2009 was mainly due to the effectiveness of the vaccination program [34].

Louisirirotchanakul el al. [35] published a study that evaluated two multiplex NAT assays (the Chiron PROCLEIX ULTRIO test individually and the Roche Cobas Taqscreen MPX test in pools of 6) for screening blood donors at NBC in 2011. A total of 486,676 seronegative donations were screened by NAT, and 175 NAT-positive/HBsAg-negative (NAT-yield) donors detected by either assay were identified. The index donations of these donors were further tested for HBsAg, anti-HBs, total anti-HBc, HBeAg, anti-HBe, and HBV viral loads. These donors were followed up for ID-NAT by both assays, HBsAg, HBeAg, anti-HBe, anti-HBs, total anti-HBc, anti-HBc IgM, and ALT monthly for up to 13 months. They found from testing the index donations that 113 (64.6%) of these donors were anti-HBc-positive, consisting of 82 (46.9%) donors who were positive for anti-HBc only and 31 (17.7%) donors who were positive for anti-HBc and anti-HBs. On following up 72 (41.1%) donors, they found that the majority of the NAT-yield donors (66.7%; 48 donors) were OBI donors. Nineteen (26.4%) donors had a primary, acute infection. This group included 15 (20.8%) window-period donors who had negative serology at their initial donation but tested positive for HBsAg (seven donors) or both anti-HBc and anti-HBs (eight donors) during follow-up. The remaining four (5.5%) donors were in the acute, late stage; they initially tested positive only for anti-HBc but showed a high anti-HBs titer (>1,000 IU/L) at follow-up, indicating a primary anti-HBs response. Three (4.2%) donors had reinfection or breakthrough infection based on positive anti-HBs only at index donation, but anti-HBc seroconversion and rising anti-HBs titer on follow-up. Two (2.7%) donors had unconfirmed HBV infection based on discrepant NAT results at index donation, which were negative on follow-up. The authors concluded that due to the majority of NAT-yield donors being OBI donors, NAT testing was essential for screening blood donors in Thailand.

Discussion

The literature review provided the HBV screening algorithms in blood donors and the prevalence of OBI and anti-HBc-positive donors from Thailand and countries in various continents of the world, including Asia, Europe, Africa, and the Americas, fulfilling the general and specific objectives of the article. The conclusions of each paper are summarized in Table 3.

**Table 3:** Summary of the conclusions of the reviewed papers anti-HBc, antibody to hepatitis B core antigen; anti-HBe, antibody to hepatitis B e antigen; anti-HBs, antibody to hepatitis B surface antigen; dHBV, hepatitis B virus discriminatory assay; DNA, deoxyribonucleic acid; HBeAg, hepatitis B e antigen; HBsAg, hepatitis B surface antigen; HBV, hepatitis B virus; ID-NAT, individual donation nucleic acid testing; IgM, immunoglobulin M; IU/L, international units per liter; IU/mL, international units per milliliter; MP-NAT, minipool nucleic acid testing; NAT, nucleic acid testing; NBC, National Blood Centre; OBI, occult hepatitis B virus infection; TT-HBV, transfusion-transmitted hepatitis B virus infection; VNRD, voluntary non-remunerated donor

Title	Conclusions	Bibliography
Prevalence of occult hepatitis B virus infection among the blood donors in Golestan province: cross-sectional study	Of the HBsAg- and HBV DNA-negative samples, 384 (8.9%) were anti-HBc-positive, of which 302 (78.65%) were anti-HBs-positive: 39.07% with a titer of 10–100 IU/mL and 60.93% with a titer of 100–600 IU/mL. Thirty-nine (0.90%) samples were anti-HBc-positive only. Confirmatory testing for anti-HBc should be done in isolated anti-HBc samples to avoid deferring blood donors based on false-positive anti-HBc results; anti-HBc testing with ID-NAT should be performed in areas with moderate prevalence of HBV.	[26]
Detection of occult hepatitis B and window period infection among blood donors by individual donation nucleic acid testing in a tertiary care center in South India	Five (0.0205%) donors were HBV DNA-positive and HBsAg-negative: four samples were positive for anti-HBc and classified as true OBI donors, of whom three (75%) were anti-HBs-positive and all four were HBeAg-negative; one sample was negative for both anti-HBc and anti-HBs and was classified as a window period donor. Anti-HBc is only appropriate in low endemicity settings (<2% anti-HBc positivity) due to the discard rates, and it cannot detect window period infection; NAT-based screening for all HBsAg-negative donations should be implemented in endemic areas instead.	[27]
Occult hepatitis B virus infection among Egyptian blood donors	In total, 525 (16.6%) HBsAg-negative donors were positive for anti-HBc, of whom 64% were anti-HBs-positive: 27.8% with a titer of 10–99 IU/L, 38.4% with a titer of 100–999.9 IU/L, and 33.8% with a titer of ≥1,000 IU/L. Confirmatory testing confirmed 90.6% of anti-HBc-positive donors, giving a prevalence of 14.2% for anti-HBc positivity among blood donors. HBV DNA was detected in 17.2% of anti-HBc-positive donors, of whom 49% had a viral load <200 IU/mL. Eleven recipients received blood from anti-HBc confirmed positive donors, but all were negative for all HBV markers on follow-up at 3–6 months: nine of these donors were HBV DNA-negative and two were HBV DNA-positive.	[28]
Evaluation of an individual-donation nucleic acid amplification testing algorithm for detecting hepatitis B virus infection in Chinese blood donors	Of the HBsAg-negative donations, 2,033 (0.17%) were multiplex ID-NAT-positive: 30.9% were HBV discriminatory assay (dHBV) positive, 31.1% of dHBV-negative samples were anti-HBc-positive and anti-HBs-negative, and 81.8% of multiplex ID-NAT-positive, dHBV-negative donations from anti-HBc-positive donors had negative anti-HBs or an anti-HBs titer of less than 100 IU/L. ID-NAT repeat testing to increase the probability of identifying low-viral-load window period and OBI donations is more practical than including anti-HBc in the screening algorithms.	[29]
Evaluating the cost-effectiveness of low-level HBV DNA screening in occult hepatitis B infection donors: A study from Shandong Blood Center, China	A total of 0.031% (69/220,445) of HBsAg-negative donors were HBV DNA-positive by MP-NAT: 82.09% were anti-HBc-positive and 37.31% were anti-HBs-positive, comprising 8.96% with a titer of 10–20 IU/L, 17.91% with a titer of 100–200 IU/L and 4.48% with a titer of 200–500 IU/L. Among HBsAg-negative and MP-NAT-negative donors, 18.95% (909/4,797) were anti-HBc-positive, of whom 1.22% were positive on further ID-NAT testing, giving an OBI detection rate of 0.22% (1.22 × 0.1895). Anti-HBc should be implemented and ID-NAT should be performed only on anti-HBc-positive donors in resource-limited settings; plasma-containing products from anti-HBc-positive donors with low or negative anti-HBs should be discarded; anti-HBs and anti-HBc should be tested in recipients, and recipients with low or negative anti-HBs should not be transfused with plasma-containing products from anti-HBc-positive donors.	[16]
Serologic and molecular profile of anti-HBc-positive blood bank donors in an area of low endemicity for HBV	Of the HBsAg-negative, HBV DNA-negative, anti-HBc-positive donors, 85.7% (209/244) were anti-HBs-positive: 14.8% with a titer of ≥10 to <100 IU/L and 70.9% with a titer of ≥100 IU/L. All were HBeAg-negative (100%) and 66.5% were anti-HBe-positive. A statistically significant association was found between anti-HBe-positive individuals and a strongly positive anti-HBs titer of ≥100 IU/L (p = 0.026). To prevent the high discard rate in times of blood shortages, measures should be taken to facilitate the use of these blood units in HBV low endemic areas.	[30]
Detection of an acute asymptomatic HBsAg negative hepatitis B virus infection in a blood donor by HBV DNA testing	A blood donor tested positive for HBV in a pool of 96 donations while being serology negative: one week later, total anti-HBc and anti-HBc IgM were positive; 45 days later, total anti-HBc was positive only; 3 months later, anti-HBs became positive with a titer of 12 IU/L; and 2 years later, the anti-HBs titer was 10 IU/L. HBsAg and HBV DNA were negative in all four follow-up samples, and HBeAg and anti-HBe were negative at all visits. This represented an asymptomatic, self-limiting acute HBV infection with consecutive immunity in an immunocompetent adult. HBV DNA screening with a sensitive assay is recommended so that not only acute HBV infection but also OBI would be detected. Anti-HBc screening would also reduce the residual risks in chronic isolated anti-HBc-positive donors, who usually have a low viral load and in whom HBV DNA therefore failed to be detected.	[31]
Blood donor screening in the Netherlands: Universal anti-HBc screening in combination with HBV nucleic acid amplification testing may allow discontinuation of hepatitis B virus antigen testing	Single marker yield was 5 MP-NAT-only, 0 HBsAg-only, and 20 anti-HBc-only donors with confirmed or probable recent HBV infection. A total of 1,067 potentially infectious donors who were at increased risk for OBI were identified by high or “true” anti-HBc reactivity. Based on the two-strike policy of anti-HBc reactive donor deferral, 350 anti-HBs-negative donors with one-time anti-HBc false reactivity were reentried. HBsAg is redundant in the presence of universal anti-HBc screening because it did not intercept infectious donations from donors with recent infection or OBI. Due to the low infectious dose of HBV, switching from MP-NAT plus anti-HBc screening to ID-NAT will reduce TT-HBV.	[32]
Occult hepatitis B virus infection and blood transfusion	The prevalence rates of anti-HBc-positive blood donors vary among countries from 0.23% to 17.28%, with an HBV DNA positivity rate of 0–15% among anti-HBc-positive-only donors, and the prevalence of OBI in blood donors is 8.55 per 1 million donations. OBI is transfusion transmissible, but the risk is lower than transmission during the window period. HBV NAT is more suitable than anti-HBc for screening in HBV endemic areas because it can detect window period infections.	[21]
Hepatitis B virus infection among first-time blood donors in Italy: Prevalence and correlates between serological patterns and occult infection	A total of 100 donors (0.32%) were positive for both HBsAg and anti-HBc, 2 donors (0.01%) for HBsAg alone, and 2,593 donors (8.3%) for anti-HBc alone. Among the last group, 0.55% were HBV DNA-positive (OBI), 47.3% were anti-HBs-positive, 39.4% were anti-HBs and anti-HBe-positive, 10.4% were anti-HBc-positive-only, and 2.9% were anti-HBe-positive (the highest HBV DNA positivity rate, 7.02%). Anti-HBs titers were >3 to <10 IU/L in 5.4%, 10–100 IU/L in 30.7%, and >100 IU/L in 63.9%. The frequency of OBI among Italian first-time blood donors is 1 in 2,599 donors. HBsAg and HBV DNA screening and HBV vaccinations are essential to reduce the risk of TT-HBV.	[33]
Hepatitis B and hepatitis C virus in Thai blood donors	All blood donations at the NBC have been made by VNRDs since 1985. All donations have been screened by serology since 1986. NAT was implemented in 2006. The HBV vaccination program was implemented in 1992. HBV vaccine coverage was 98.3% in 2008. The drastic change of HBV prevalence in Thai first-time blood donors, from 7.14% in 1988 to 2.63% in 2009, was mainly due to the effectiveness of the vaccination program.	[34]
Occult hepatitis B virus infection in Thai blood donors	Of the NAT-yield donors, 113/175 (64.6%) were anti-HBc-positive: 82 donors (46.9%) were positive for anti-HBc only and 31 donors (17.7%) were positive for both anti-HBc and anti-HBs. On follow-up of the donors for up to 13 months, 66.7% were OBI donors, 20.8% were window period donors, 5.5% were acute, late stage donors, 4.2% had reinfection or breakthrough infection, and 2.7% had unconfirmed HBV infection. Due to the majority of NAT-yield donors being OBI donors, NAT testing was essential for screening blood donors in Thailand.	[35]

OBI donors have HBV DNA in their liver, and sometimes it is detected in their serum by screening using NAT. The prevalence of OBI is estimated to range from 8.55 per 1 million donations to 1 in 2,599 first-time donors. The clinical data are clear that OBI donations are transfusion-transmissible, although the risk is lower than window period donations. Therefore, once HBV DNA is detected in a donor’s serum, he or she should be permanently deferred. HBV DNA is detected in 0-15% of anti-HBc-positive-only donors.

Window period donors can have HBV screening test results similar to OBI donors, i.e., HBsAg-negative and HBV DNA-positive. Blood units from window period donors are more infectious than donations from OBI donors. Anti-HBc screening has a disadvantage of its inability to detect window period donors.

Anti-HBc-positive-only donors may have false positivity in their anti-HBc test result or may have been infected with HBV in the past. The prevalence rates of anti-HBc-positive-only donors vary from 0.23% to 17.28% depending on the country. For the false-positive category, a confirmatory anti-HBc test should be done to avoid discarding non-infectious blood units and unnecessary donor deferral. For the later HBV-infected category, the protective anti-HBs should be further tested. Donors with a high titer of anti-HBs and/or low anti-HBc (titer <1:32 in Japan) can be allowed to donate blood. A high titer of anti-HBs is defined as more than 200 IU/L in Japan, the Netherlands, and the recommendations of the SaBTO of the UK, or more than 100 IU/L in Germany, Turkey, and Austria. The rationale behind this policy is that the protective anti-HBs with high titer can prevent the transmission of HBV to the recipients. Studies showed that high percentages of these anti-HBc-positive-only donors were anti-HBs-positive, including 78.95%, 64%, 85.7%, and 86.7% of donors in Iran, Egypt, Brazil, and Italy, respectively. Moreover, the majority of these donors have high titers of anti-HBs using the cut-off of anti-HBs >100 IU/L, including 60.93%, 72.2%, 70.9%, and 63.9% of anti-HBc and anti-HBs-positive donors in Iran, Egypt, Brazil, and Italy, respectively. Therefore, approximately half of anti-HBc-positive donors would be allowed to donate and their donation would be saved from discarding if the cut-off of anti-HBs titer more than 100 IU/L is used.

For Thailand, an upper-middle income, HBV endemic country, the HBV screening algorithm in use at NBC, which accounts for almost half of all blood donations in the country consisting of HBsAg and ID-NAT, is appropriate. Anti-HBc is only appropriate for screening in low endemicity settings (<2% anti-HBc positivity) due to the high discard rates; therefore, in Thailand, which still has a high HBV prevalence in the population that was born before the universal HBV vaccination program was implemented in 1992, i.e., 2.1% in those aged 41-50, 2.2% in those aged 51-60, and 4.3% in those aged over 60, anti-HBc screening is unsuitable. Moreover, a study conducted in Thai blood donors at NBC showed that 64.6% of NAT-yield donors were anti-HBc-positive; therefore, 35.4% of HBV DNA-positive donors were anti-HBc-negative and could be missed if the screening was done by anti-HBc and the less sensitive MP-NAT. Furthermore, the majority (66.7%) of the NAT-yield donors were OBI donors; therefore, the sensitive ID-NAT rather than the less sensitive pooled NAT is appropriate to intercept these OBI donors who can have very low HBV DNA in the plasma but can transmit HBV to the recipients if high plasma-containing blood products were transfused. Lastly, 20.8% of the NAT-yield donors were window period donors, which could be missed if the screening was done by anti-HBc, and their donations, which are more infectious than those from OBI donors, could be mistakenly used for transfusion.

## Conclusions

TTIs remain a risk of blood transfusion, even though much lower than before, due to three methods pf prevention: blood donor selection, laboratory testing, and PRT. TT-HBV can still occur due to OBI donors, anti-HBc-positive only donors, and window period donors. HBV screening algorithms to detect these HBV-infected donors differ from country to country based on the prevalence of HBV in the general population of the country and the resources of the country. In non-endemic, resource-abundant countries, the HBV screening algorithms usually involve HBsAg, anti-HBc, and MP-NAT. In non-endemic, resource-limited countries, the HBV screening algorithms usually involve HBsAg and MP-NAT. In endemic, resource-abundant countries, the HBV screening algorithms usually involve HBsAg and ID-NAT. In endemic, resource-limited countries, the HBV screening algorithms usually involve HBsAg only, but one study from China suggested incorporating ID-NAT repeat testing, and another study from the same country suggested universal anti-HBc screening and ID-NAT only in anti-HBc-positive donors.

In conclusion, each blood center should develop its own screening algorithms for transfusion-transmissible infections based on local prevalence, available resources, and scientific research. In regions with universal anti-HBc screening, HBsAg testing may offer limited additional yield and could eventually become redundant, with a potential shift toward ID-NAT due to the low infectious dose of HBV. For the NBC in Thailand, ID-NAT remains essential for detecting OBI and window-period donors. Since broad anti-HBc screening is not practical for Thailand's donor population, the current NBC algorithm combining HBsAg and ID-NAT remains the most appropriate strategy.
